# Accurate prediction of functional effects for variants by combining gradient tree boosting with optimal neighborhood properties

**DOI:** 10.1371/journal.pone.0179314

**Published:** 2017-06-14

**Authors:** Yuliang Pan, Diwei Liu, Lei Deng

**Affiliations:** 1 School of Software, Central South University, Changsha, China; 2 Shanghai Key Laboratory of Intelligent Information Processing, Shanghai, China; Tianjin University, CHINA

## Abstract

Single amino acid variations (SAVs) potentially alter biological functions, including causing diseases or natural differences between individuals. Identifying the relationship between a SAV and certain disease provides the starting point for understanding the underlying mechanisms of specific associations, and can help further prevention and diagnosis of inherited disease.We propose PredSAV, a computational method that can effectively predict how likely SAVs are to be associated with disease by incorporating gradient tree boosting (GTB) algorithm and optimally selected neighborhood features. A two-step feature selection approach is used to explore the most relevant and informative neighborhood properties that contribute to the prediction of disease association of SAVs across a wide range of sequence and structural features, especially some novel structural neighborhood features. In cross-validation experiments on the benchmark dataset, PredSAV achieves promising performances with an AUC score of 0.908 and a specificity of 0.838, which are significantly better than that of the other existing methods. Furthermore, we validate the capability of our proposed method by an independent test and gain a competitive advantage as a result. PredSAV, which combines gradient tree boosting with optimally selected neighborhood features, can return reliable predictions in distinguishing between disease-associated and neutral variants. Compared with existing methods, PredSAV shows improved specificity as well as increased overall performance.

## Introduction

Single amino acid variants (SAVs) are single-base changes that result in amino acid changes of the encoded protein [1]. With the rapid development of sequencing and genomic analysis technologies, substantial SAVs between individuals have been uncovered. The 1000 Genomes project [2] and recent sequencing of whole human genomes [3–6] have provided a large number of single-nucleotide polymorphisms (SNPs), insertions, deletions and structural variants in humans. Among these variations, SAVs are recognized as the most common type in the human genome [7, 8], and some are often closely related to particular diseases [9–11]. According to the previous studies, SAVs may be responsible for the initiation or progression of cancer through aberrant proteins [12]. And the amino acid change can affect, for example, protein stability, interactions and enzyme activity, thereby leading to disease. Therefore, the identification of whether a SAV is neutral or disease-associated is playing an increasingly important role in understanding the underlying mechanisms of specific SAV-disease associations and developing treatment strategies for diseases.

However, experimentally determining the SAV-disease relationship of such a large number of variants is time-consuming and costly. Accurate computational approaches are vital for analysis the relationship between SAV and disease. Current prediction methods typically employ machine learning algorithms [13–16] such as neural networks [17], random forests(RF) [18] and support vector machines (SVMs) [19], and a large variety of properties, including amino acid sequence features [20], position-specific scoring matrices, residue-contact network features and 3-D structure information. This includes methods such as SIFT [21, 22], SNAP [23], Polyphen2 [24], FunSAV [25] and SusPect [26]. SIFT uses sequence homology to predict phenotypic effect based on the assumption that amino acid variants in the evolutionarily conserved regions are more likely to have functional effects [21, 22]. SNAP [23] combines multiple sequence analysis methods with neural networks to predict the functional effects of variants. Polyphen2 [24] predicts the functional impact of a variant by a Naive Bayes classifier trained using sequence, phylogenetic and structural information. FunSAV utilizes a two-stage random forest with a large number of sequence and structural properties to discriminate the SAV-disease links [25]. Yates et al. combine sequence and structural features to build an SVM classifier named SusPect to predict disease-SAV associations [26].

**Fig 1:**
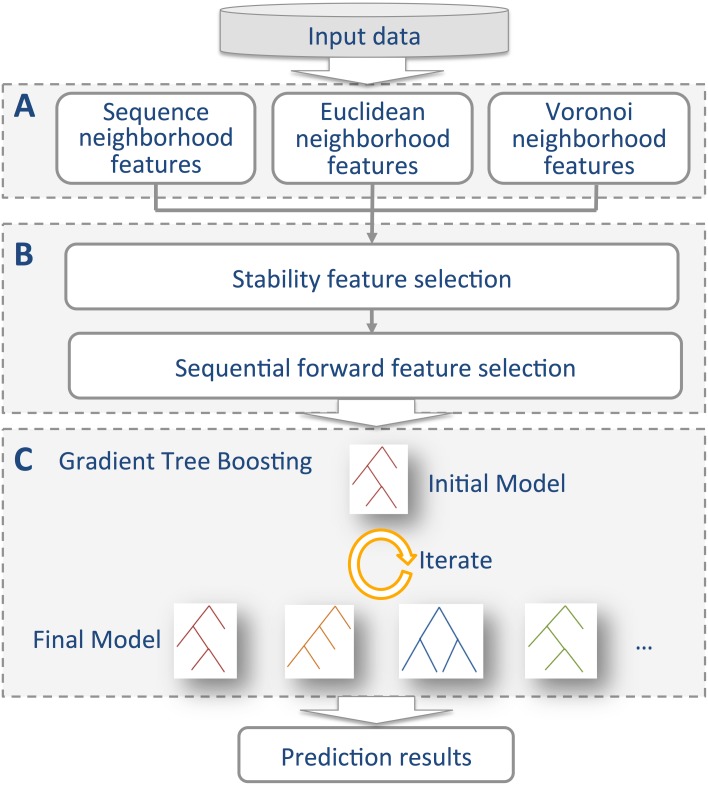
The framework of PredSAV. (A) Feature representation. A total of 1521 sequence, Euclidean and Voronoi neighborhood features are initially generated. (B)Two-step feature selection. Stability selection is used as the first step. We select the top 152 features with score larger than 0.2. The second step is performed using a wrapper-based feature selection. Features are evaluated by 5-fold cross-validation with the GTB algorithm. (C) Prediction model. Gradient boosted trees are finally built for prediction.

In this work, we develop a novel approach, termed as PredSAV, to predict the phenotypic effects of SAVs by using the Friedman’s gradient tree boosting [27, 28] algorithm. PredSAV combines both sequence neighborhood features and structural neighborhood features describing not only the properties of the target residue but also the target residue’s neighborhood environment. PredSAV uses a efficient two-step feature selection method to eliminate uninformative properties, which in turn improves the performance and helps to build faster and more cost-effective models. Extensive comparisons of PredSAV with other existing tools on the benchmark dataset and another independent dataset show that PredSAV significantly outperforms the existing state-of-the-art methods, and illustrate the effectiveness and advantage of the proposed approach. The framework of PredSAV is shown in Fig 1.

## Materials and methods

### Datasets

The dataset, taken from Wang and Song [25], are mainly gathered from the UniProt [29] human sequence variations and the Ensemble human variation database [30]. Disease-associated variants are obtained from the UniProt human sequence variations and non-Mendelian disease variants without any OMIM entry [31] are removed. Neutral variants are extracted from the Ensemble human variation database. All the SAVs emanate from the HapMap project [32]. We remove the redundant homology sequences at the sequence similarity of 40% by using CD-HIT [33]. Then a BLAST search [34] is used to map the remaining sequences to PDB structures [35]. Structures with resolutions lower than 2.5Å are removed. Ambiguity or invalid SAV-disease associations and neutral variants are deleted. Obsoleted PDB structures, such as 1VJJ and 2HR0, are removed. Eventually, the dataset consists of 670 proteins containing 1006 disease-associated and 963 neutral variants. A total of 816 disease-associated and 776 neutral variants are randomly selected as the benchmark dataset and the rest are used as the independent dataset including of 190 disease-associated and 187 neutral variants.

### Performance evaluation

We evaluate the performance of the proposed method using 5-fold cross-validation and several widely used measures. These measures include sensitivity (SEN/Recall), specificity (SPE), precision (PRE), F1-score (F1), accuracy (ACC), the Matthew’s correlation coefficient (MCC) and the area under the ROC curve (AUC).

$$\begin{array}{c} SEN=TP/(TP+FN) \end{array}$$ (1)

$$\begin{array}{c} SPE=TN/(TN+FP) \end{array}$$ (2)

$$\begin{array}{c} PRE=TP/(TP+FP) \end{array}$$ (3)

$$\begin{array}{c} F1=\frac{2\times Recall\times Precision}{Recall+Precision} \end{array}$$ (4)

$$\begin{array}{c} ACC=\frac{TP+TN}{TP+TN+FP+FN} \end{array}$$ (5)

$$\begin{array}{c} MCC=\frac{TP\times TN-FP\times FN}{\sqrt{\left(TP+FP)(TP+FN)(TN+FP)(TN+FN\right)}} \end{array}$$ (6)

### Features extraction

In our experiment, a wide variety of sequence and structure features are generated for predicting the phenotypic effects of SAVs. Several novel structural features, including residue-contact network features, solvent exposure features and structural neighborhood features, are calculated. The details of these features are listed as follows.

#### Sequence features

A large number of sequence features are calculated: 1) position-specific scoring matrices (PSSMs) [34]; 2) predicted solvent accessibility using the SSpro and SSpro8 programs [36]; 3) predicted native disorder by DISOPRED [37]; 4) the dScore that represents the difference between the PSIC [38] scores for the wild type amino acid residue and mutant amino acid residue, calculated by PolyPhen2 [24]; 5) predicted disorder in proteins by DisEMBL [39]; 6) the local structural entropy of a particular residue is computed by LSE [40]; 7) the eight physicochemical properties for each amino acid are obtained from the AAindex database [41]; 8)BLOSUM62 [42] was used to count the relative frequencies of amino acid and their substitution probabilities; 9) solvent accessible surface area, secondary structure and local backbone angles generated by SPIDER2 [43]; 10) predicted the relative solvent accessibility of protein residues by the ACCpro and ACCpro20 from the SCRATCH package [36]; 11)evolutionary conservation scores calculated based on PSSM [34] and Jensen-Shannon divergence [44, 45].

#### Structure features

Structural features, including secondary structure, four-body statistical pseudo-potential, solvent accessibility and exposure features, are calculated as candidate features for SAV phenotype prediction. We used DSSP [46] to calculate the secondary structure features, including hydrogen bonds, solvent-accessible surface area, C_*α*_ atom coordinates and backbone torsion angles. The four-body statistical pseudo-potential is based on the Delaunay tessellation of proteins [47]. Delaunay tessellation is a effective way to define the structural neighbors of a target protein. The potential is defined as follows:

$$\begin{array}{c} \begin{array}{c} Q_{ijkl}^{\alpha }=log\;\left[\frac{f_{ijkl}^{\alpha }}{p_{ijkl}^{\alpha }}\right] \end{array} \end{array}$$ (7)

where i, j, k, and l are the residue identities of the four amino acids in a Delaunay tessellation of the target protein. Each residue is represented by a central point among the atoms in the residue. $f_{ijkl}^{\alpha }$ is the observed frequency of the residue composition (*ijkl*) in a tetrahedron of type *α* over a set of protein structures. $p_{ijkl}^{\alpha }$ is the expected random frequency.

Energy scores including side-chain energy score, residue energy, conservation, interface propensity, combined1 score, combined2 score and relative solvent accessibility are calculated by using ENDES [48]. Two combined energy scores are also used. The combined1 score is a combination of residue energy, conservation and interface propensity scores. The combined2 score is an optimized combination weights of the three features to get the best prediction of single residue.

Solvent-accessible related features have been shown to be very useful in identifying SAV-disease association [49–51]. We use NACCESS [52] and NetSurfP [53] to calculate solvent accessibility for the protein structures, respectively. The NACCESS program is used to calculate the absolute and relative solvent accessibilities of all atoms. For NetSurfP, the absolute and relative surface accessibility, Z-fit score and secondary structure are computed based on the homology proteins obtained from PSI-BLAST search.

Solvent exposure features, include the coordination number (CN), number of *C*_*β*_ atoms in the upper Half-Sphere (HSEBU), number of *C*_*β*_ atoms in the lower Half-Sphere(HSEBD) and residue depth (RD), are calculated by HSEpred [54] and the hsexpo program [55]. The hsexpo program uses protein structure information, while the HSEpred uses sequence information to predict these features.

#### Residue-contact network features

Residue-residue contact networks have bee proved to be very beneficial for analyzing and predicting SAV-disease associations [56]. If the distance between the centers of two residues in a structure are within 6.5Å, an edge exists between the two residues in the network. We use NAPS [57] to compute the residue-residue contact network properties, which describe the local environment of the target variant in the network, including betweenness, closeness, coreness, degree, clustering coefficient, eigenvector centrality, eccentricity and average nearest neighbor degree.

#### Structural neighborhood features (SNF)

Conventional features usually describe only the properties of the current residue itself, cannot represent the real environment well, and thus are insufficient to predict functional effects of SAVs with high precision. Here, we calculate two types of structural neighborhood features (SNF) based on Euclidean distance and Voronoi diagram [58–60], respectively. Surrounding residues located within a sphere of the radius of 5*Å* are defined as the Euclidean neighborhood of the central amino acid. The Euclidean distance is computed between any heavy atoms of the surrounding residues and that of the central amino acid. The score of a specific feature *i* for the central residue *r* regarding the neighbor *n* is defined as follows:

$$\begin{array}{c} F_{i}\left(r,n\right)=\left\{\begin{array}{c} \begin{array}{c} \text{the}\;\text{score}\;\text{of}\;\text{feature}\;i\;\text{for}\;\text{residue}\;r\;\text{if}\;|r-n|\geq 1\;and\;d_{r,n}\leq 5Å, \end{array}\\ \;0\;\;\text{otherwise} \end{array}\right. \end{array}$$ (8)

where *d*_*r*,*n*_ is the minimum Euclidean distance between residue *r* and residue *n*. The Euclidean neighborhood feature of the central residue *r* is defined as:

$$\begin{array}{c} EN_{i}\left(r\right)=\sum_{n=1}^{m}F_{i}\left(r,n\right), \end{array}$$ (9)

where *m* is the total number of Euclidean neighbors.

Voronoi neighborhood features are calculated based on Voronoi diagram/Delaunay triangulation. For a 3-D protein structure, individual atoms are devided into Voronoi polyhedra by Voronoi tessellation partition. In the Voronoi diagram (Delaunay triangulation), a pair of residues are defined as Voronoi neighbors if there exists at least one common Voronoi facet between heavy atoms of each residue. The Qhull package [61] is used to calculate Voronoi/Delaunay polyhedra. For the target residue *r* and its Voronoi neighbors *n* {*n* = 1, …, *m*}, the Voronoi neighborhood property of the feature *i* is defined as:

$$\begin{array}{c} VD_{i}=\sum_{n=1}^{m}P_{i}\left(n\right),\; \end{array}$$ (10)

where *P*_*i*_(*n*) is the score of the residue feature *i* for neighbor *n*.

#### Feature encoding with neighborhood properties

For each sample, a combination of 1,287 (117*11) sequence neighborhood features, 117 Euclidean neighborhood features and 117 Voronoi neighborhood features are calculated. The sequence neighborhood features is generated by applying a sliding window of size 11 to incorporate the evolutionary information from upstream and downstream neighbors in the protein sequence.

### Feature selection

The feature selection method improves the performance by removing some redundant features in high-dimensional data [62–65]. In this study, we propose a two-step feature selection approach to select the most important features for predicting the phenotypic effects of SAVs. First, we assess the feature elements using the stability selection [66] calculated by the RandomizedLasso package in the scikit-learn [67]. The idea of stability selection is that a feature selection algorithm is employed on subsample datasets and subsample features. The selection results are merged after repeating a certain number of times. Stronger features have higher scores (close to 1), while weaker features have scores close to 0. The score represents the importance of an individual feature for correctly predicting an SAV-disease association. Here, we select the top 152 features with the score larger than 0.2.

The second step is performed using a wrapper-based feature selection method. The features are evaluated by 5-fold cross-validation with the GTB (gradient tree boosting) algorithm, and correlation features are added by sequential forward selection (SFS). In the SFS scheme, features are sequentially added to a null feature set till an optimal feature subset is obtained. Each added feature is the one whose add maximizes the performance of the classifier. This stepwise feature selection process continues until the AUC score no longer increased. As a result, a set of 44 optimal features are selected as the final optimal feature set.

### Gradient tree boosting algorithm

The Gradient Tree Boosting (GTB) [27, 28] is an effective machine learning algorithm that can be utilized for both classification and regression problems. In this study, GTB is implemented under the PredSAV framework as shown in Fig 1 and the prediction of the phenotypic effect of single amino acid variants could be considered as a binary classification problem. For a large number of given input feature vectors ***χ***_*i*_ (***χ***_*i*_ = {*x*_1_, *x*_2_, …, *x*_*n*_}, *i* = 1, 2, …, *N*) with labels *y*_*i*_ (*y*_*i*_*ϵ*{−1, +1}, *i* = 1, 2, …, *N*, where “-1” represents neutral variant and “+1” denotes disease-associated variant), the purpose of the GTB algorithm is to build an effective classifier to predict whether a variant is disease-associated or neutral. The GTB algorithm is shown in Algorithm 1.

**Algorithm 1** Gradient Tree Boosting Algorithm

**Input**:

 Data set: *D* = {(***χ***_1_, *y*_1_), (***χ***_2_, *y*_2_), …, (***χ***_*N*_, *y*_*N*_)}, ***χ***_*i*_*ϵ****χ***, ***χ*** ⊆ ***R***, *y*_*i*_*ϵ*{−1, +1}; loss function: *L*(*y*, Θ(***χ***)); iterations = M;

**Output**:

1: Initialize $\Theta_{0}\left(\chi \right)=arg\;min_{c}\sum_{i}^{N}L\left(y_{i},c\right);$

2: **for** *m* = 1 to M **do**

3: Compute the negative gradient as the working response

$r_{i}=-{\left[\frac{\partial L(y_{i},\Theta (\chi_{i}))}{\partial \Theta (\chi_{i})}\right]}_{\Theta (\chi )=\Theta_{m-1}(\chi )},i=\{1,\ldots ,M\}$

4: The input ***χ***_*i*_ is adapted to the classification model *r*_*i*_ by Logistic function and get the estimate ***α***_*m*_ of *βh*(***χ***; ***α***)

5: Get the estimate *β*_*m*_ by minimizing *L*(*y*_*i*_, Θ_*m*−1_(***χ***_*i*_) + *βh*(***χ***_*i*_; ***α***_*m*_))

6: Update Θ_*m*_(***χ***) = Θ_*m*−1_(***χ***) + *β*_*m*_*h*(***χ***; ***α***_*m*_)

7: **end for**

8: **return** $\tilde{\Theta }\left(\chi \right)=\Theta_{M}\left(\chi \right)$

In the algorithm, the variable *iterations* = *M* should be initialized. The logistic function is used as the loss function, which is defined as:

$$\begin{array}{c} L(y,\Theta (x))=log(1+exp(-y\Theta (\chi ))), \end{array}$$ (11)

where *y* is a real class label of variants and Θ(*χ*) is a decision function. The decision function is initialized by the following equation.

$$\begin{array}{c} \Theta_{0}\left(\chi \right)=arg\;min_{c}\sum_{i=1}^{N}L\left(y_{i},c\right), \end{array}$$ (12)

where *N* is the number of SAVs in the benchmark dataset. Then, GTB constructs m different classification trees *h*(***χ***, ***α***_1_), *h*(***χ***, ***α***_2_), …, *h*(***χ***, ***α***_*m*_) from a number of benchmark datasets. The addictive function Θ_*m*_(*x*) can be defined as:

$$\begin{array}{c} \Theta_{m}\left(\chi \right)=\Theta_{m-1}\left(\chi \right)+\beta_{m}h\left(\chi ;\alpha_{m}\right) \end{array}$$ (13)

Above, the *β*_*m*_ and ***α***_*m*_ are a weight and vector of parameters for the *m*-th classification tree *h*(***χ***, ***α***_*m*_), respectively. In order to minimize the loss function *L*(*y*, Θ_*m*_(***χ***)), the weight of *β*_*m*_ and the parameter of ***α***_***m***_ need to be iterated from *m* = 1 to *m* = *M*. In the third step, the negative gradient *r*_*i*_ as the working response by the following formula:

$$\begin{array}{c} r_{i}=-{\left[\frac{\partial L(y_{i},\Theta \left(\chi_{i}\right))}{\partial \Theta (\chi_{i})}\right]}_{\Theta \left(\chi \right)=\Theta_{m-1}\left(\chi \right)},i=1,\ldots ,M \end{array}$$ (14)

Then, the weight of *β*_*m*_ and the parameter of ***α***_*m*_ for the *m*th iteration can be defined as:

$$\begin{array}{c} \left(\beta_{m},\alpha_{m}\right)={arg\;min}_{\beta ,\alpha }\;\sum_{i=1}^{N}L\left(y_{i},\Theta_{m-1}\left(x_{i}\right)+\beta h\left(x_{i};\alpha_{m}\right)\right) \end{array}$$ (15)

However, we do not directly calculate the above equation. In the fourth step, the input ***χ***_*i*_ is adapted to the classification model *r*_*i*_ by Logistic function and get the estimate ***α***_*m*_ of *βh*(***χ***_*i*_; ***α***). Therefore, we can obtain

$$\begin{array}{c} \alpha_{m}=arg\;min_{\alpha }\;\sum_{i=1}^{N}\frac{1}{1+e^{r_{i}h\left(\chi_{i},\alpha \right)}} \end{array}$$ (16)

In the fifth step, the estimate parameter of *β*_*m*_ is obtained by minimizing the log loss function *L*(*y*, Θ(*χ*)).

$$\begin{array}{c} \begin{array}{c} \begin{array}{cc} min & \;L(y_{i},\Theta_{m-1}\left(\chi_{i}\right)+\beta h\left(\chi_{i};\alpha_{m}\right))\\ & =min\;log(1+exp\left(-y_{i}\left(\Theta_{m-1}\left(\chi_{i}\right)+\beta h\left(\chi_{i};\alpha_{m}\right)\right)\right)) \end{array} \end{array} \end{array}$$ (17)

Then, in the sixth step, a new addictive function Θ_*m*_(***χ***) is updated by in the eq (8). Finally, we obtain a classification function Θ_*M*_(***χ***) and a useful GTB model $\tilde{\Theta }\left(\chi \right)$ as follows:

$$\begin{array}{c} \tilde{\Theta }\left(\chi \right)=\Theta_{M}\left(\chi \right) \end{array}$$ (18)

We use a grid search strategy to select the optimal parameters of GTB with 5-fold cross-validation on the benchmark dataset. The optimized number of trees of the GTB is 2000. And the selected depth of the trees is 3. The rest use the default parameters.

The source code and data are available at http://www.leideng.org/PredSAV/.

## Results and discussion

### Benefits of the two-step feature selection

The selection of informative attributes is critically important for building effective and accurate classification models. In total 1521 sequence, Euclidean and Voronoi neighborhood features are initially generated. We apply a two-step feature selection method, consisting of stability selection and sequential forward selection. Stability selection is used as the first attribute selection step for two reasons. First, stability selection can address the difficult variable selection problem with markedly improved error control and structure estimation, especially for high-dimensional problems. Second, stability selection depends little on the chosen initial regularisation and can reduce the risk of overfitting [66]. To assess the utility of the stability selection method, we evaluate the performance by incorporating the GTB classifier with selected attributes that correspond to different cutoffs of stability selection scores. As shown in Table 1, when the number of selected features decreases from 1521 to 152 (the cutoff increases from 0 to 0.2), the highest accuracy of 81.3% is yielded. The other measurements (SEN, SEP, PRE, MCC and AUC) are observed as 0.810, 0.817, 0.823, 0.628 and 0.896, respectively. We select the top 152 features (stability score > 0.2) as the input of the next sequential forward selection step. A set of 44 optimal features is finally selected with the highest AUC score of 0.908. The results of selected features show ∼2% and ∼5% increase in AUC and MCC over the initial features, respectively.

**Table 1:** Performance of selected attributes with the two-step feature selection method. The first column lists different cutoffs of stability selection scores.

Features	Number	ACC	SEN	SEP	PRE	MCC	AUC
All features	1521	0.804	0.802	0.807	0.815	0.608	0.881
score>0.1	263	0.808	0.804	0.813	0.812	0.610	0.886
score>0.15	191	0.808	0.809	0.807	0.815	0.616	0.892
score>0.2	152	0.813	0.810	0.817	0.823	0.628	0.896
score>0.25	112	0.810	0.809	0.813	0.819	0.622	0.893
score>0.3	93	0.809	0.810	0.806	0.815	0.618	0.890
score>0.35	84	0.810	0.809	0.808	0.814	0.618	0.889
score>0.4	69	0.809	0.808	0.809	0.814	0.615	0.888
Final optimal features	44	0.826	0.814	0.838	0.839	0.651	0.908

**Fig 2:**
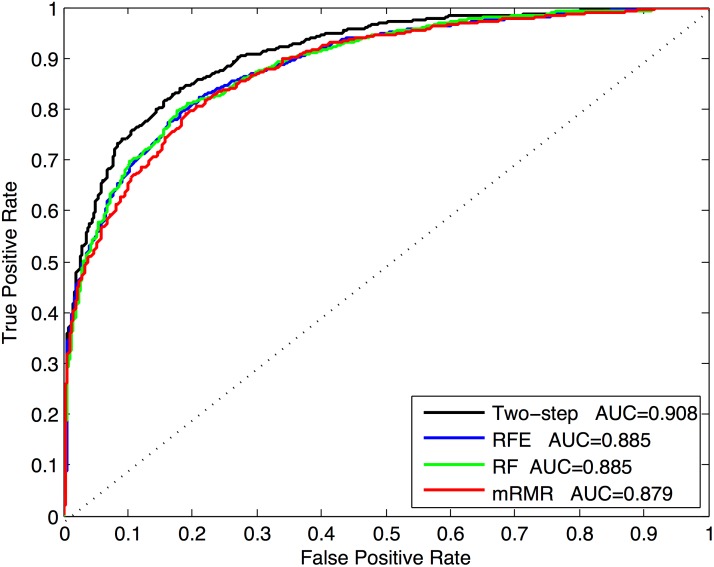
ROC curves of our two-step algorithm and other three existing feature selection methods.

We compare the proposed two-step feature selection method with three widely used feature selection methods: random forests(RF), maximum Relevance Minimum Redundancy(mRMR) [68] and Recursive Feature Elimination(RFE) [69]. The experiment is based on the benchmark dataset with 5-fold cross-validation. Fig 2 shows the ROC curves of the four feature selection methods. The results are shown in Fig 2. Our two-step feature selection approach obtains the best performance. The results indicate that our two-step feature selection algorithm, which is a composite approach combining the merits of both stability selection and sequential forward selection, can substantially boost the prediction performance with less computational expense and lower risk of overfitting.

### Feature importance

**Fig 3:**
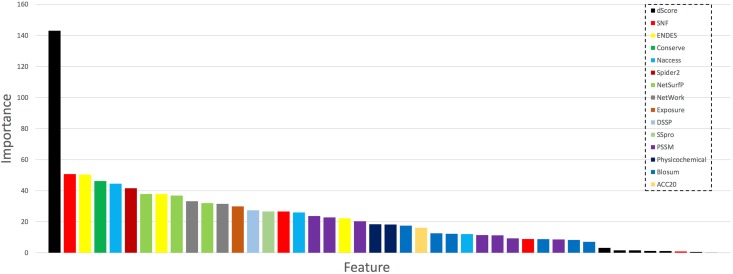
The relative importance and ranking of the optimal feature group, as evaluated by the gradient tree boosting. The bar represents the importance score of the corresponding feature group.

The feature importance of these features are calculated by using the gradient tree boosting method. The relative importance and rankings of the optimal features are shown in Fig 3 and Table 2. Among them, the feature with the highest score (>100) is the dScore feature calculated by PolyPhen2. The dScore represents the difference between the PSIC scores of the wild type amino acid residue and mutant amino acid residue. Another important feature that have not been found useful in previous studies is structural neighborhood features (Euclidean and Voronoi). We find that structural neighborhood features achieve the second highest score compared with other features, suggesting that structural neighborhood features are critical in distinguishing disease-associated SAVs from neutral SAVs. Solvent accessibilities are also found to be useful in SAVs phenotype prediction. Many solvent accessibility related features, including the solvent accessibility feature calculated by NACCESS, NetSurfP, DSSP, solvent exposure features, ACC20 and the SSpro score, are important and contributive. The results suggest that sequence and structural neighborhood features complement each other quite well and thus collectively make a contribution to the performance enhancement.

**Table 2:** Rankings of feature importance for the optimal selected features. SN, EN and VN represent sequence neighborhood, Euclidean neighborhood and Voronoi neighborhood, respectively. The numbers in the brackets denote the positions in the sliding window for sequence neighborhood features.

Rank	Feature name	Type	Rank	Feature name	Type
1	dScore(6)	SN	23	Flexibility parameter in physicochemical(5)	SN
2	dScore	VN	24	BLOSUM(H)(7)	SN
3	RA in ENDES(4)	SN	25	ACC20 in SCRATCH(11)	SN
4	Conservation score(1)	SN	26	BLOSUM(N)(3)	SN
5	Non-polar ABS in Naccess(6)	SN	27	BLOSUM(L)(4)	SN
6	ASA in SPIDER2(6)	SN	28	Main-Chain ABS in Naccess(9)	SN
7	Z-fit score in Netsurfp(10)	SN	29	PSSM(C)(9)	SN
8	RA in ENDES(8)	SN	30	PSSM(S)(10)	SN
9	ASA in Netsurfp(2)	SN	31	PSSM(C)(1)	SN
10	Betweenness in NetWork(9)	SN	32	ACC	EN
11	ASA in Netsurfp(6)	SN	33	BLOSUM(L)(6)	SN
12	Closeness in NetWork(6)	SN	34	hydrophobic moment in physicochemical(3)	SN
13	HSEBU in HSEpred(7)	SN	35	BLOSUM(T)(7)	SN
14	KAPPA in DSSP(7)	SN	36	BLOSUM(C)(4)	SN
15	SSpro in the SCRATCH(7)	SN	37	dScore(11)	SN
16	combined2 in ENDES(4)	SN	38	dScore(1)	SN
17	Total-Side REL in Naccess(9)	SN	39	dScore(9)	SN
18	PSSM(N)(10)	SN	40	dScore(3)	SN
19	PSSM(I)(6)	SN	41	dScore(2)	SN
20	PROPENSITY in ENDES(5)	SN	42	dScore	EN
21	PSSM(G)(1)	SN	43	dScore(10)	SN
22	Flexibility parameter in physicochemical(11)	SN	44	dScore(7)	SN

### Gradient tree boosting improves predictions

**Fig 4:**
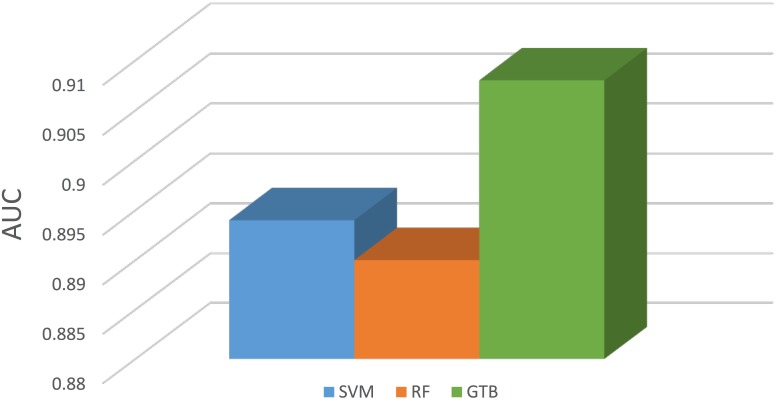
Comparison of the AUC value of the the three methods using 5-fold cross-validation on the benchmark dataset.

PredSAV uses Gradient Tree Boosting (GBT) to build the final model with the 44 optimal features. We compare GBT with Support Vector Machine (SVM) and Random Forests (RF), which are well known to perform fairly well on a variety of tasks. Fig 4 shows the AUC scores of GTB and other machine learning methods on the final optimal feature set. GTB, SVM and RF achieve AUC values of 0.908, 0.894 and 0.890, respectively. Comparing with the other methods, the GTB model can improve the prediction preformance. Note that the GBT algorithm is implemented with scikit-learn [67] in this study.

### PredSAV outperforms other predictors

To evaluate the performance of the proposed PredSAV, six existing SAVs prediction methods, including FunSAV [25], Polyphen2 [24], SusPect [26], SIFT [21], SNAP [23] and nsSNPAnalyzer [70], are evaluated on the benchmark dataset.

**Fig 5:**
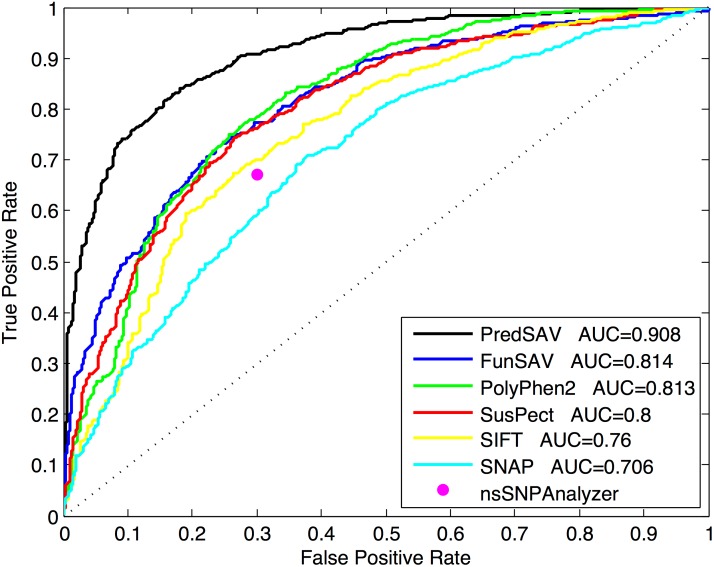
The ROC curves of seven classifiers on the benchmark dataset.

Table 3 and Fig 5 show the detailed results of comparing our method with the existing methods. Overall, our approach shows dominant advantage over the existing methods in six metrics: ACC, SPE, PRE, F1, MCC and AUC. When comparing the AUC score with that of the existing classifiers, FunSAV (0.814), Polyphen2 (0.813), SusPect (0.800), SIFT (0.760) and SNAP (0.706), our PredSAV classifier (0.908) shows greater improvement by 9%, 9%, 11%, 14% and 20%, respectively. For the remaining measurements ACC, SPE, PRE, F1 and MCC, we can observe similar increases. Especially, the specificity of PredSAV is significant higher than other methods (increased by 10%), which suggests that it has better performance detecting true negatives and may help for reducing experiment cost. Only in SEN, PredSAV is lower than PolyPhen2 and SNAP (0.855 and 0.866 for SNAP and PolyPhen2, respectively). We can observe that PredSAV gains a balanced sensitivity and specificity (0.814 and 0.838, respectively), suggesting that PredSAV has better balance of prediction accuracy between disease-associated and neutral SAVs.

**Table 3:** Prediction performance of PredSAV classifiers in comparison with six other prediction tools on the benchmark dataset.

Method	ACC	SEN	SEP	PRE	F1	MCC	AUC
**PredSAV**	**0.826**	0.814	**0.838**	**0.839**	**0.826**	**0.651**	**0.908**
FunSAV	0.749	0.762	0.736	0.753	0.757	0.508	0.814
PolyPhen2	0.732	**0.866**	0.590	0.690	0.768	0.476	0.813
SusPect	0.723	0.653	0.798	0.775	0.709	0.455	0.800
SIFT	0.697	0.699	0.695	0.707	0.703	0.394	0.760
SNAP	0.635	0.855	0.395	0.605	0.709	0.284	0.706
nsSNPAnalyzer	0.712	0.745	0.661	0.774	0.759	0.401	-

### Performance confirmed for independent test

**Fig 6:**
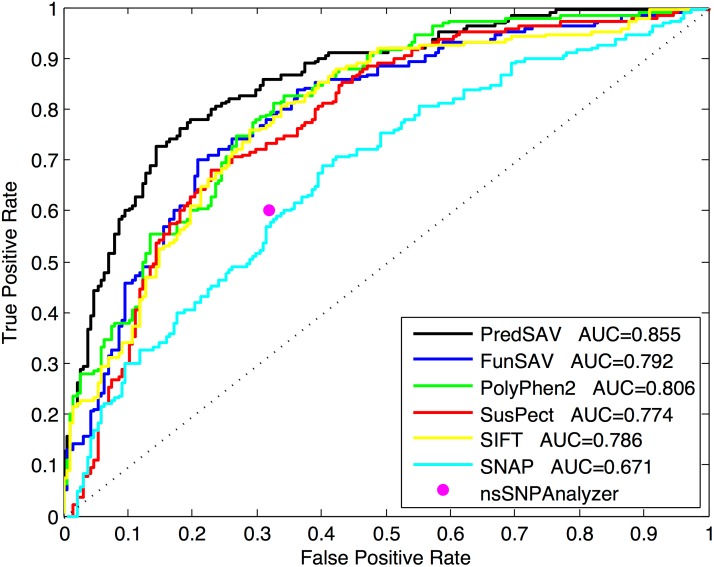
The ROC curves of seven classifiers on the independent test dataset.

We also validate the performance of PredSAV on the independent test dataset to avoid over-optimistic performance estimates. Results of the independent test are presented in Table 4, which indicate marked improvements for all the performance measures except SEN comparing PredSAV with the exiting methods. Fig 6 shows the ROC curves. The ROC curves indicate the trade-off between the amounts of true positives (TP) and false positives (FP) generated by the classifiers. We observe that PredSAV produces higher true positive rates of prediction across most of the false positive rates. Overall, these observations suggest that the performance of our PredSAV approach is superior to that of the state-of-the-art approaches.

**Table 4:** Prediction performance of PredSAV classifiers in comparison with six other prediction tools on the independent test dataset.

Method	ACC	SEN	SEP	PRE	F1	MCC	AUC
**PredSAV**	**0.790**	0.780	**0.802**	**0.800**	**0.789**	**0.581**	**0.855**
FunSAV	0.731	0.769	0.701	0.679	0.721	0.480	0.792
PolyPhen2	0.727	0.868	0.583	0.679	0.762	0.471	0.806
SusPect	0.716	0.684	0.749	0.734	0.708	0.434	0.774
SIFT	0.729	0.774	0.684	0.714	0.742	0.460	0.786
SNAP	0.594	**0.879**	0.305	0.562	0.686	0.225	0.671
nsSNPAnalyzer	0.639	0.705	0.550	0.677	0.691	0.258	-

### Case study

**Fig 7:**
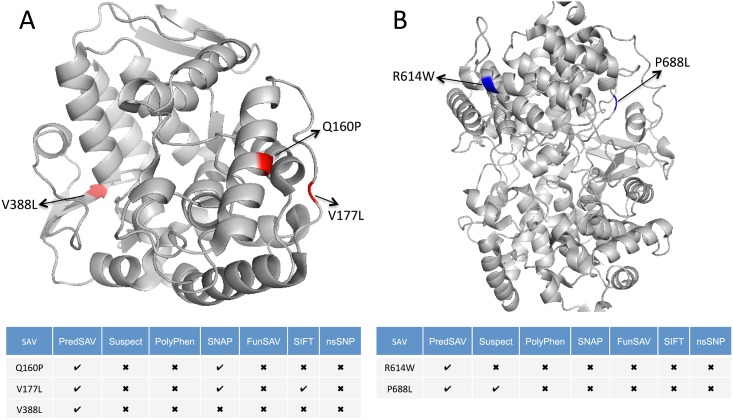
Prediction examples of the functional effects of SAVs in two proteins by PredSAV and other methods. Red color denotes disease-associated variants while blue color represents neutral variants. (A) and (B) represent proteins PAH (PDB ID: 1J8U, chain A) and LSS (PDB ID: 1W6K, chain A), respectively. 3-D structures are rendered using PyMol [75].

To further illustrate the effectiveness of PredSAV, we present examples by comparing predictions for variants that are difficult to classify with commonly applied methods. The enzyme phenylalanine hydroxylase (PAH, PDB ID: 1J8U, chain A) [71, 72] is responsible for the conversion of phenylalanine to another amino acid, tyrosine. PAH works with a molecule called tetrahydrobiopterin (BH4) to carry out this chemical reaction. The majority of mutations in PAH result in deficient enzyme activity and cause hyperphenylalaninemia. Some cause phenylketonuria (PKU), others cause non-PKU hyperphenylalaninemia, while still others are silent polymorphisms. As shown in Fig 7A, three PKU-associated SAVs, Q160P (dbSNP:rs199475601), V177L (dbSNP:rs199475602) and V388L (dbSNP:rs62516101), are colored in red. This example illustrates how PredSAV combines gradient tree boosting with optimal neighborhood features to provide better predictions. PredSAV (TP = 3) correctly identified all the three disease-associated variants, compared to Suspect (TP = 0), PolyPhen (TP = 0), SNAP (TP = 2), FunSAV (TP = 0), SIFT (TP = 1) and nsSNP (TP = 0).

Another example is lanosterol synthase (LSS, PDB ID: 1W6K, chain A) [73, 74], which catalyzes the cyclization of (S)-2,3 oxidosqualene to lanosterol, a reaction that forms the sterol nucleus. Through the production of lanosterol may regulate lens protein aggregation and increase transparency. The variants R614W (dbSNP:rs35785446) and P688L (dbSNP:rs17293705) in LSS are neutral substitutions. From Fig 7B, we can see that PredSAV can predict the neutral SAVs successfully, while other existing methods result in almost completely wrong results (except Suspect in P688L). This suggests that PredSAV has the highest specificity, which is desirable for many biological applications since it allows researchers to identify a short list of SAVs for targeted phenotype studies.

## Conclusion

In this study, we present a novel approach named PredSAV for producing reliable predictions in distinguishing between effect and neutral variants. To be able to do this, we first extract a very large collection of imformative and complementary features, including sequence, structure, network and neighborhood features that describe the local environments proximal to the centered variant and neighboring residues. A two-step feature selection approach, which combines stability selection and sequential forward selection, is utilized to select an optimal subset of features within a reasonable computational cost, and thus improves the prediction performance and reduces the risk of overfitting. Importantly, the use of gradient tree boosting algorithm further attains higher levels of prediction accuracy. We evaluate the PredSAV method with both cross-validation and independent test, and the results indicate that the proposed PredSAV is able to identify disease-associated SAVs with higher overall performance, especially in terms of specificity, when compared with other existing approaches.

A limitation of PredSAV is that it requires the 3D protein structure, which may limit its broader application. However, with the increasing solved protein structures, protein homology modeling projects [76] and predicted 3D structures [77], it is expected that PredSAV can be used as a powerful tool to prioritize the disease-associated variants and help towards the phenotypic effect annotation of these targets.

As for future work, we will explore more efficient features to further improve the performance and learn from other methods [78–82] to provide a a web-server for the method proposed in this paper.

## Supporting information

S1 File The disease-associated and neutral variant data used in these experiments. (ZIP)
